# A Contribution to the Study of the Infectiousness of Milk from Tuberculous Cows and as to the Value of the Tuberculin-test1Translated from the Zeitzchrift für Hygiene und Infectionskrankheiten (xxxi., 1) of May 26, 1899, for the Journal of Comparative Medicine and Veterinary Archives.

**Published:** 1899-09

**Authors:** Lydia Rabinowitch, Walter Kempner

**Affiliations:** The Institute for Infectious Diseases and the Pathological Institute of the Veterinary High School in Berlin; The Institute for Infectious Diseases and the Pathological Institute of the Veterinary High School in Berlin


### SELECTION.

### A CONTRIBUTION TO THE STUDY OF THE INFECTIOUSNESS OF MILK FROM TUBERCULOUS COWS AND AS TO THE VALUE OF THE TUBERCULIN-TEST.[1]

BY DR. LYDIA RABINOWITCH AND DR. WALTER KEMPNER.

(*From the Institute for Infectious Diseases and the Pathological Institute of the Veterinary High School in Berlin.*)

NUMEROUS bacteriological examinations of milk made during the last decade have furnished experimental proof of the presence of tubercle bacilli in market milk in a greater or less percentage of samples. For example, the Berlin market milk was shown by Obermüller to contain tubercle bacilli in 61 per cent., and by Petri only in 14 per cent. of the samples tested on animals. The researches made by one of us (L. Rabinowitch) in Berlin in 1897 showed that of twenty-eight samples, of varying origin, seven of them, or 28 per cent., contained tubercle bacilli. The milk was centrifuged and the sediment and cream were mixed and injected intraperitoneally into guinea-pigs.[2]

A further question remained to be answered—that is, *whether the milk containing tubercle bacilli came only from cows that were afflicted with severe general tuberculosis involving the udder, or also from animals in which there was no udder-tuberculosis and only more or less severe lung lesions.* A large number of investigations relating to this question are published. In 1880 Bollinger produced inoculation tuberculosis in experimental animals with the milk from a cow that had tuberculosis of the udder, and also with milk from a tuberculous cow in which there was no perceptible disease of the udder. Other investigations were made under Bollinger's supervision by May and Stein. May inoculated with the milk from six tuberculous cows and obtained a positive result in but one case, in which the milk came from a cow in which the disease was highly developed and involved the udder. Stein made a series of experiments consisting of fourteen intraperitoneal injections with milk from cows suffering with tuberculosis in different stages, and obtained four positive results. In these four cases the cows furnishing milk were highly tubercular, but none had diseased udders. Bang has

[1] Translated from the Zeitzchrift für Hygiene und Infectionskrankheiten (xxxi., 1) of May 26, 1899, for the Journal of Comparative Medicine and Veterinary Archives.

[2] The details of this investigation are shown in a table in the original article.

made numerous investigations in this direction since the year 1884, and, altogether, from sixty-three highly tuberculous cows has found the milk from nine (14 per cent.) to be infectious; although, according to his view, the milk of the majority of tuberculous cows is not dangerous, it can be infectious without visible disease of the udder in case the cow in question is in an advanced stage of tuberculosis.

Herschberger also concludes, from his investigations, that the milk can be infectious without tubercular disease of the udder, even when there is but little disease of the lungs. However, in advanced generalized tuberculosis or in udder-tuberculosis the infectiousness of the milk is greatest. In twenty cases Herschberger obtained eleven positive results (55 per cent.) from his inoculations. Ernst has examined one hundred and fourteen samples of milk from thirty-six tuberculous cows without infected udders, and has found the milk infectious in 28.57 per cent. In 50 per cent. of these cases the infectiousness was determined by inoculation alone. In 35.7 per cent. the tubercle bacilli were also found by microscopical examination.

Smith and Schroeder examined six tuberculous cows without discoverable udder-disease, and obtained positive results in two cases (32 2 per cent.), both by inoculation and by microscopical examination. Schroeder also examined thirty-one cows in which tuberculosis had been recognized either by clinical examination or by reaction to tuberculin; unfortunately, the details in regard to the tuberculin test are not furnished. Disease of the udder could not be found in any of these cases. In only two cases (6.5 per cent.) could positive inoculation results be obtained from the injection of the centrifugalized milk. Both of these cases were in advanced stage of disease, and in one of them this was proved by a post-mortem examination. Delepine, by inoculation, proved the infectiousness of the milk of two out of six cows that had reacted to tuberculin and also presented physical evidences of tuberculosis. In both of these cases there was well-marked udder-tuberculosis, and this diagnosis was confirmed by microscopical examination. Of seven tuberculous cows that were not tested with tuberculin the milk was infectious in only one case, and in this cow disease of the udder was doubtful. Delepine has also examined the milk from twenty-four cows that were suspected of having tuberculosis, and of these ten had clearly diseased and nine suspected udders. The other five cows had healthy udders.

| Milk from udders. | | Producing tuberculosis. Actual No. | | Percentages. | |
|---|---|---|---|---|---|
| A. Certainly diseased | 10 | 5 | 6 | 50.0 | 30.5 |
| B. Probably diseased | 9 | 1 | | 11.1 | |
| C. Healthy | 5 | 0 | | 0 | |
| Total | 24 | 6 | | | |

From this little table we see that the infectiousness of the milk was proved only when it came from cows with diseased or suspected udders.

Some of the authors above named, and also Johne, Woodhead, McFadyean and others have made microscopical examinations of milk for tubercle bacilli, and, having found acid-resisting bacteria, have considered the milk tuberculous. We shall return later, in connection with our investigations, to the subject of the unreliability of this tinctorial evidence.

The results obtained by the various authors may be tabulated as follows:

| Authors. | No. of cows under experiment. | No. of times tubercle bacilli were found in the milk. | Percentage of positive results. |
|---|---|---|---|
| May | 6 | 1 | 16.6 |
| Stein | 14 | 4 | 28.5 |
| Bang | 63 | 9 | 14.0 |
| Hirschberger | 20 | 11 | 55.0 |
| Ernst | 36 | 10 | 28.5 |
| Smith and Schroeder | 6 | 2 | 33.2 |
| Schroeder | 31 | 2 | 6.5 |
| Delepine | 37 | 9 | 24.3 |
| Nocard | 54 | 3 | 5.5 |
| Rabinowitsch and Kempner | 15 | 10 | 66.6 |

It is thus shown by experiments on animals that milk from tuberculous cows is infectious in from 6 to 55 per cent. of the cited cases. The greatest infectiousness was possessed by the milk of cows afflicted with advanced general tuberculosis or tuberculosis of the udder. On the other hand, there are isolated instances in which milk was infectious from cows presenting but slight evidence of tuberculosis. As opposed to this experience, we have the repeatedly declared view of Nocard, who holds that milk is infectious only when there is well-marked clinical disease or udder-infection. He says that the milk from cows presenting disease of the udder that can only be detected by microscopical examination is not very dangerous. Nocard has himself inoculated with the milk from fifty-four cows with generalized tuberculosis, and has obtained positive results in but three cases (5.5 per cent), and in these the udder was diseased.

In the reports on the infectiousness of the milk of tuberculous cows—a matter of such extraordinary hygienic importance—we fail in most of those cited to find exact statements regarding the clinical condition of the diseased animals, the result of the tuberculin test, the post-mortem condition, etc. This information is absolutely necessary for the settlement of the question above stated. If we admit that the milk from cows with advanced or with udder-tuberculosis is not sold (which scarcely conforms with the facts), or, if sold, only under the necessary precautions, such as Pasteurization or sterilization, then, in the light of our present knowledge, the above question may be formulated as follows: *Does milk contain tubercle bacilli, first, in beginning tuberculosis without discoverable disease of the udder, and, second, in latent tuberculosis that can be detected only by the tuberculin reaction?* The second point has not been considered sufficiently since the epoch-making discovery of tuberculin.

The Minister of Agriculture ordered that experiments should be made at the Veterinary High School in Berlin, under the direction of Prof. Schuetz, for the purpose of testing the curative value of various substances in tuberculosis of cattle. It thus happened that a large number of tuberculous cows were purchased, and, at the suggestion of Profs. Koch and Schuetz, we have endeavored, through the use of these cows, to answer the questions formulated above. Fifteen cows, all of which had reacted to tuberculin, were used for this investigation. The examination of the milk occurred, excepting in one cow (No. 15), about three months after the tuberculin-test. The milk was drawn without contamination direct into sterilized flasks. The cows were well milked out, and only the second part of the milk was taken for investigation three or four hours after milking. This, as in the case of our earlier milk examinations, was centrifuged, and guinea-pigs were inoculated intraperitoneally with a mixture of the fat layer and the sediment.

Very little information is available as to the technique of the milking and centrifuging practised in earlier examinations of the milk from tuberculous cows. Obermüler has called attention to the importance of centrifuging in his investigations of the market milk of Berlin.

Usually, quantities of from 3 to 5 c.c. were injected. If the animals died from peritonitis, another injection of from 1 to 2 c.c. was made from the same sample. Separate microscopical examinations were made from the fat layer and from the sediment of every sample. Preparations were stained by the Ziehl-Neelsen method, some with the fat removed and some without. We thus obtained the astonishing result that in eleven of fifteen samples acid-resisting bacteria were found. However, in only two cases, in which the preparation contained numerous acid-resisting rods, was it possible by reference to the form and positions to identify them with reasonable certainty as tubercle bacilli. The correctness of this view was confirmed by animal experiments. By inoculation, tuberculosis was established ten times. In other cases it was not possible to differentiate between the true tubercle bacilli and the tubercle-bacilli-like rods discovered by Koch and described by Petri and Rabinowitsch. If we had attempted to decide between tuberculosis and pseudotuberculosis by microscopical examination we would have erred on both sides, as was shown by the results of inoculation. The possibility that both forms of bacteria may occur together in milk is not excluded.

In view of the recent investigations of Moeller, who has obtained cultures of acid-resisting bacteria from timothy grass, which is used so much as fodder, we need not be surprised at the frequent occurrence of tubercle-bacilli-like rods in milk and butter. Since in our experiments the milk was drawn aseptically, we must assume that these are excreted through the udder. Moeller has also isolated a similar acid-resisting bacterium from the intestinal contents of cattle and from cow manure, so that we must doubt previous reports as to the frequent occurrence of tubercle bacilli in the manure of tuberculous cattle. At the meeting of naturalists in 1897 Eber said: "The most important source of tubercle bacilli in milk is the soiling of milk with particles of manure which may be carriers of virulent tubercle bacilli. One should recall that the bronchial secretion of cattle containing tubercle bacilli is not expelled through the mouth, as in man; but, on account of the fact that cattle are permanently swallowing, it passes into the intestinal canal and is expelled with the feces." Although we may admit the truth of Eber's views as to the discharge of sputum containing bacilli with the feces, we are constrained to oppose his statement as to the frequent contamination of milk with particles of feces infected with tubercle bacilli. While Eber, without this theory, "found it impossible to explain the frequent appearance of real tubercle bacilli in the milk, in spite of the comparatively small number of cases of generalized and udder-tuberculosis among cattle," we will show that our samples of milk drawn aseptically from cows but slightly tuberculous and with healthy udders were frequently infectious.

Turning to the microscopical examination of milk for tubercle bacilli, we are of the opinion that in the light of our present knowledge and of the results that we have ourselves obtained, evidence based upon this form of examination cannot be admitted as bearing on the question of the infectiousness of milk.

As to our inoculation experiments with the milk from fifteen cows, all of which had reacted to tuberculin, we have obtained positive results in ten cases—that is, 66.6 per cent.

If we exclude the milk from cow No. 5, which was a yellowish, gelatinous fluid, and caused fatal peritonitis in all of the animals inoculated with it, then we will have a still higher percentage of positive results (71.4 per cent.), which is very much in advance of those obtained by previous authors. (See table.) In reference to the clinical condition of the ten cows that furnished infectious milk we submit the following facts obtained by Prof. Eggling's examination :

1. Only one cow (No. 12) presented clinical evidence of udder-tuberculosis.

2. In one cow (No. 9) udder-tuberculosis was present, but was revealed only by microscopical examination.

3. In cows Nos. 1, 6, and 11 advanced generalized tuberculosis was detected. The udders of these cows were not tuberculous, but by histological examination were found to be in a state of chronic interstitial inflammation.

4. Cow No. 4 was slightly tuberculous.

5. In cow No. 10 it was only upon the second and third examinations that beginning tuberculosis could be detected.

6. When cow No. 8 was examined the first time crepitant râles were heard; upon both of the following examinations no signs of tuberculosis were present.

7. Cow No. 2 did not show any signs of tuberculosis upon either of the three examinations, and cow No. 14 also presented no evidence of tuberculosis.

### REPORT ON THE EXAMINATION OF THE FIFTEEN TUBERCULOUS COWS.

These cows were all tested with tuberculin, with the results mentioned below. They were also examined clinically by Prof. Eggling three times. The first examination was made July 3d, the second October 18th, and the third December 5th.

Cow No. 1. About twelve years old. Tested with tuberculin

July 3d. Temperature reaction 2° C. First examination revealed rough breathing, cough, swelling of the supramammary lymphatic glands. Second examination: Rough breathing, cough, swelling of the lymphatic glands of the udder, cachexia, diarrhœa, presumably intestinal tuberculosis. This cow died October 18, 1898. Post-mortem examination revealed acute and chronic tubercular lesions of the lungs, abscesses between the second stomach and diaphragm, encapsulated abscesses of the liver, chronic interstitial inflammation of the udder. The milk contained tubercle bacilli.

Cow No. 2. About twelve years old. Tested with tuberculin July 3d; reaction 2.4° C. First examination: No evidence of tuberculosis. Second examination: No evidence of tuberculosis. Third examination: No evidence of tuberculosis; condition of nutrition fair. The milk contained tubercle bacilli.

Cow No. 3. About five years old. Tested with tuberculin July 18th; temperature reaction, 2° C. First examination: Dyspnœa, rough breathing, and râles. Second examination: Dyspnœa, râles, harsh breathing, crepitant and friction râles in the posterior portions of both lungs. Third examination: Rough coat, frequent cough, rough breathing, occasionally râles, bronchial murmur on the right side, dulness on the left. No tubercle bacilli were found in the milk.

Cow No. 4. About five years old. Tested with tuberculin July 18th; temperature reaction, 2.7° C. First examination revealed rough breathing and a few râles. Second examination: Rough breathing. Third examination: Condition of nutrition fair, respiration accelerated and labored, rough breathing, with a few râles, cough. The milk contained tubercle bacilli.

Cow No. 5. About eight years old. Tested with tuberculin July 18th; temperature reaction, 2° C. First examination: Rough breathing, a few râles. Second examination: Rough breathing. Third examination: Condition of nutrition good, nothing abnormal found. This cow died on January 16, 1899. Post-mortem examination revealed acute miliary tuberculosis of the lungs, tuberculosis of the mediastinal, bronchial, and mesenteric lymphatic glands; miliary tubercles in the liver and on the intestines, and chronic interstitial inflammation and abscesses in the udder. The milk was yellow and gelatinous, and contained numerous pus-cells and cocci.

Cow No. 6. About seven years old. Tested with tuberculin July 25th; temperature reaction, 1.7° C. First examination:

Cough, shrill breathing on the right side. Second examination: Frequent cough and many crepitant râles on both sides. Third examination: Smooth coat, condition of nutrition better, frequent rough cough, numerous dry and moist râles, harsh breathing on both sides. This cow was killed on June 8, 1899. Post-mortem examination showed tuberculosis of the lungs, with ulcerating cavities, cheesy areas in the liver and in the mesenteric glands, also in many other lymphatic glands; localized chronic interstitial inflammation of the udder. The milk contained tubercle bacilli.

Cow No. 7. About eight years old. Tested with tuberculin July 25th; temperature reaction, 1.6° C. First examination: Frequent cough, friction-sounds on the right side, dulness on the left side. Second examination: Dyspnœa, rough breathing, slight dulness on the left side toward the root of the lung, frequent cough, and a few crepitant râles on the right side, well back. Third examination: Rough coat, frequent cough. No tubercle bacilli were found in the milk.

Cow No. 8. About five years old. Tested with tuberculin July 25th; temperature reaction, 1.7° C. First examination: Râles in the right side. Second examination: No evidence of tuberculosis. Third examination: Smooth coat, good condition, nothing abnormal. The milk contained tubercle bacilli.

Cow No. 9. Between five and six years old. Tested with tuberculin August 1st; temperature reaction, 1.7° C. First examination: Dyspnœa, rough breathing, frequent cough, dry râles, especially on the upper right side; bronchial murmuring in the middle of the right side; dulness. Third examination: Advanced cachexia, dyspnœa, inability to stand, sobbing breathing, many dry râles, dulness on both sides. This cow died December 6, 1898. Post-mortem examination revealed wide-spread old and fresh tuberculous lesions of the lungs, on the intestines, uterus, pleuræ, peritoneum, and in numerous lymphatic glands; chronic interstitial inflammation and tuberculosis of the udder. The milk contained tubercle bacilli.

Cow No. 10. Between five and six years old. Tested with tuberculin August 1st; temperature reaction, 1.9° C. First examination: No symptoms. Second examination: Rough and labored breathing, cough intensified, vesicular murmur. Third examination: Rough coat, frequent and rough breathing. The milk contained tubercle bacilli

Cow No. 11. Tested with tuberculin August 1st; temperature reaction, 2.8° C. First examination: Frequent cough, crepitant râles. Second examination: Few râles, cough, rough breathing. Third examination: Poor nutritive condition increased, vesicular murmur, occasional cough, few crepitant râles. This cow was killed January 8, 1899. Post-mortem examination revealed widespread old and fresh tubercular lesions in the lungs, cheesy alterations on the liver and various lymphatic glands. Part of the udder showed chronic interstitial inflammation, and both sides were catarrhal and purulent. The milk contained tubercle bacilli.

Cow No. 12. Between six and seven years old. Tested with tuberculin August 7th; temperature reaction, 2.2° C. First examination: Harsh breathing, coughing. Second examination: Coughing. Third examination: Nutritive condition better, frequent breathing, occasional cough, râles, tuberculosis of the udder. The milk contained tubercle bacilli.

Cow No. 13. Reacted to tuberculin, but presented no clinical evidence of tuberculosis. No tubercle bacilli were found in the milk.

Cow No. 14. Reacted to tuberculin, but presented no clinical evidence of tuberculosis. The milk contained tubercle bacilli.

Cow No. 15. Tested with tuberculin December 16th; temperature reaction, 1.6° C. Physical examination on January 3, 1899: Accelerated respiration, rough breathing, a few râles, and dulness over the posterior portion of the right lung, some tympanitis. No tubercle bacilli were found in the milk.

These results show that our investigations differ from those of earlier authors, not only in respect to the higher percentage of infected milk, but also in respect to the clinical condition of the cows furnishing the milk. Previous authors have found the milk to contain tubercle bacilli in most cases only where udder-tuberculosis or advanced tuberculosis was present.

From our experience we must answer the questions previously stated as follows:

*Milk may contain tubercle bacilli, first, in beginning tuberculosis without discoverable disease of the udder, and, second, in latent tuberculosis that can be detected only by the tuberculin reaction.*

As to the first point, we refer again to cows Nos. 4, 10, and 8.

As to the second point, to cows Nos. 2 and 14, that were discovered to be tuberculous only by the application of the tuberculin test. It is noticeable that cow No. 2 furnished both samples of milk, in which we could detect the tubercle bacilli with the microscope with reasonable accuracy.

As to the other five more or less diseased cows, in the milk from which we found no tubercle bacilli, we will say that we were prevented from making repeated inoculations with the milk from these animals. That such repetition is necessary is shown by the examination of the milk from cow No. 9. On August 27th we inoculated in the usual way two guinea-pigs with this milk. Butter, both salted and unsalted, was made from the same milk on that day, and was used four days later for the inoculation of four guinea-pigs. It is interesting to note that while the inoculation with the milk gave negative results, three of the four guinea-pigs inoculated with butter developed tuberculosis; one from inoculation with one sample of butter and two from inoculation with the other. Another examination of the milk from cow No. 9 furnished a positive result.

Although up to this time uncertain opinions have prevailed in reference to the infectiousness of the milk from cows that react to tuberculin and present no clinical evidence of tuberculosis, we believe, notwithstanding the small number of our experiments, that we are justified in asserting that the milk from cows that react to tuberculin must be suspected of being infectious in every case. We see, therefore, that the interesting question as to the diagnostic value of tuberculin possesses great importance, and that in addition to the clinical examination of cows and bacteriological supervision of milk (which is difficult to carry out) a tuberculin test is the most valuable measure for insuring the production of milk free from tubercle bacilli. As a result of our investigations we cannot share the opinion of Eber, who says: "The important matter in certifying to and managing herds devoted to the production of milk is not a tuberculin test (which gives no idea as to the extent of tubercular disease in the animal), but in the clinical examination and in the bacteriological supervision of milk-cows. The most important measure[1] is not the separation of the reacting from the non-reacting animals, but the most efficient exclusion from the herd of cows that show clinical evidence of tuberculosis." We must, in view of our observations on the tuberculin test, ascribe to it very much more importance in relation to the question of the infectiousness of milk than to the clinical diagnosis. We hope that the tuberculin test will receive the greatest private and public support, so that the growing dangers to public health through milk and milk-products may be counteracted.

[1] This has reference to the protection of the public from the dangers accompanying the use of the milk of tuberculous cows, and does not relate to the suppression of tuberculosis in herds. Eber holds that tuberculin testing and separation are necessary to repress tuberculosis among cattle.—*Trans.*

